# Application of Ionic Liquids in High Performance Reversed-Phase Chromatography

**DOI:** 10.3390/ijms10062591

**Published:** 2009-06-04

**Authors:** Ye Wang, Minglei Tian, Wentao Bi, Kyung Ho Row

**Affiliations:** Department of Chemical Engineering, Inha University, Incheon 402-751, Korea; E-Mails: wangye929@hotmail.com (Y.W.); feitiandezhu@hotmail.com (M.T.); bi_wentao@hotmail.com (W.B.)

**Keywords:** ionic liquids, reversed-phase high performance liquid chromatography, stationary phase, additives

## Abstract

Ionic liquids, considered “green” chemicals, are widely used in many areas of analytical chemistry due to their unique properties. Recently, ionic liquids have been used as a kind of novel additive in separation and combined with silica to synthesize new stationary phase as separation media. This review will focus on the properties and mechanisms of ionic liquids and their potential applications as mobile phase modifier and surface-bonded stationary phase in reversed-phase high performance liquid chromatography (RP-HPLC). Ionic liquids demonstrate advantages and potential in chromatographic field.

## 1. Introduction

Ionic liquids (ILs) [1], which also called room-temperature ionic liquids (RTILs) or room-temperature molten salts, are liquid at ambient temperature, and are usually composed of relatively large organic cations and inorganic or organic anions. ILs have negligible vapor pressure, good thermal stability, tunable viscosity, and primarily anion-dependent miscibility with water as well as various organic solvents. Because of these characteristic properties, ILs have been widely used in many chemical fields.

Recently, many studies have focused on ionic liquids as additives in electrochemistry [2,3], catalysis for chemical synthesis [4,5], and as organic solvents in gas chromatography (GC) and countercurrent chromatography (CCC) [6,7]. These applications of ILs are based on their good physical properties as “green” solvents.

In addition, ILs have attracted interest in the fields of separation and analysis. Ionic liquids are already applied as running electrolytes in capillary electrophoresis (CE) [8–11] and as a new class of stationary phases in gas chromatography [12–15]. Ionic liquids coated onto fused silica capillaries show a dual nature, acting as low-polarity phases with non-polar compounds as well as in the opposite manner (i.e., highly interactive and retentive) for compounds bearing strong proton-donor groups. In particular, RP-HPLC is the widest application of ILs in separation and analysis. There are many others reviews about ILs in separation techniques [16,17]. Usually ILs are used in RP-HPLC as mobile phase additives and stationary phase based functional materials.

There has been little research on ILs used in RP-HPLC, and the good properties of ILs have not been exploited completely. Overall, a review of the existing research is essential for the development of ILs in the future. The following survey was performed to evaluate the suitability of ionic liquids in separation and analysis as mobile phase additives and stationary phase functional materials. The organization of this review is as follows. The section “Mechanism of ILs” introduces their properties and provides detailed information on the mechanism of ILs used as mobile phase additives and as a stationary phase. The section “Application of ionic liquids in RP-HPLC” summarizes the theoretical background of ionic liquids applied in RP-HPLC as modifiers, followed by the preparation of IL-stationary phases. The effects of using ionic liquids and other important conditions are also discussed. The next section, “Separation of biological samples”, reviews some key examples of successful applications of ionic liquids.

## 2. Mechanism of ILs

### 2.1. Properties of ILs

The main physicochemical properties of ILs are as follows: (i) they are generally colorless liquids with relatively low viscosity; (ii) they have a very low vapor pressure and remain liquid over a temperature range of 200–300 °C in an inert atmosphere; (iii) they have a wide window of electrochemical stability, good electrical conductivity, high ionic mobility, and excellent chemical stability; and (iv) being composed of two parts, they exhibit synthetic flexibility that is not observed in single component molecular solvents. These unique properties of ILs are determined by the structure and interaction of the ions in the melt. Typically, ILs consist of nitrogen-containing organics and any array of inorganic anions (e.g., chloride, bromide, tetrafluoroborate, hexafluorophosphate), as well as ammonium, imidazolium, pyridinium, pyrrollidium, and isoquinolinium. Each of these has the probability of attaching different alkyl groups to the heterocyclic ring or quaternary cation. Depending on the type of cation under investigation and the length of the alkyl chain, the resulting ionic liquid can have a melting point above the ambient temperature (293 K) [18]. Table 2 shows the cations and anions that can be generally grouped to prepare different ILs. And Table 1 lists the physical properties of some common ionic liquids.

### 2.2. Mechanism of ILs as Additives

The most commonly used IL anions are polyatomic inorganic species, such as [PF_6_]^−^ and [BF_4_]^−^. The most prominent cations are pyridinium and imidazolium [19–29]. Liquid chromatography is defined as a separation method using a liquid mobile phase. The liquid mobile phase can be a pure solvent or in most cases, a mixture of different solvents. The very low volatility of ILs may not be of critical importance in LC.

The main problem in considering ILs as possible organic modifiers in RPLC is their very high viscosity (Table 1), which is at least one order of magnitude higher than that of methanol or acetonitrile, the two most widely used RPLC organic modifiers. Basic compounds are difficult to separate in RPLC on silica-based stationary phases due to the interaction between the cationic sites of the compounds with the anionic silanols of the stationary phase. These interactions produce peak tailing and lengthy retention of the basic compounds. Amines or divalent cations were demonstrated to be useful silanol-blocking agents [30]. Jiang suggested the addition of selected ILs as silanol screening agents in totally aqueous mobile phases in the separation of ephedrines and catecholamines [31]. The addition of 2.0–50.0 mM IL to aqueous mobile phases improved the basic compound peak shapes but these improvements were associated with changes in the retention factors. A wide variety of ILs was investigated [32] and a model involving ion-pairing and a layer of IL adsorbed on the C_18_ surface was proposed.

Kaliszan *et al*. reported significant improvement on the basic compound peak shape obtained after adding small amounts of ILs to the mobile phases [33]. The ILs had better silanol blocking activity than classical quaternary amines [34]. However, they also observed that the improvements in peak shape were associated with significant changes in retention factors. The retention factors can increase or decrease depending on the IL used. ILs become just regular salts when added as additives to aqueous solutions at low concentrations. Their specific properties, such as a low melting point, high thermal stability and extremely low vapor pressure, are lost and/or not important. As salts, ILs have a dual nature. They are obviously made by cations associated with an equal amount of anions. Both species can affect the chromatographic results.

Basic compounds most often bear positively charged amine groups in low pH mobile phases. Consequently they are retained by a combination of electrical (charge-charge) and hydrophobic interactions with the stationary phase and with ions of the mobile phase. The mixed mechanism involves ion-pairing, ion-exchange and hydrophobic partitioning. The basic compound peak position depends on the overall strength of the combined solute-stationary phase interactions. The basic compound peak shape depends on the kinetics of the interaction. In aqueous mobile phases, charge-charge interactions are usually stronger and slower than the hydrophobic interactions. In this situation, the [BMIM]^+^ cation adsorbs onto the C_18_ stationary phase without being associating with an imipramine solute. Charge–charge repulsion occurs between the [BMIM]^+^ covered stationary phase and imipramine. The retention factor is lower and the peak shape is better with imipramine being retained mainly by hydrophobic fast interactions. IL ions adsorb on the C_18_ surface and the chaotropic [BF_4_]^−^ anion can associate with the imipramine cation forming less polar ion-pairs. Consequently, the imipramine retention factor is increased and the peak shape is improved because fast hydrophobic interactions are mostly involved in the retention mechanism of the ion-pair.

Overall, the review of the use of ILs as efficient silanol screening agents in the separation of basic compounds suggests that there is no “most suitable” IL. If the basic compounds are polar and lightly retained, a polar IL additive with a strongly chaotropic anion, such as [MMIM][PF_6_], [EMIM][PF_6_] or [EMIM][ClO_4_], is recommended. With less polar and hydrophobic amines, a less polar IL additive with a cosmotropic anion, such as [BMIM][Cl] or [HMIM][Cl], is a good choice. ILs as silanol screening agents may be considered as “green” additives in that they allow for peak shape improvement and reduced retention without increasing the content of the mobile phase organic modifier [21]. However, ILs are certainly not the additive of choice with a MS detector. The non-volatility of ILs will be responsible for IL condensation and pollution in the electron-spray or atmospheric pressure-ionization sources.

### 2.3. Mechanism of ILs in the Stationary Phase

Analogous to the use of ionic liquids as mobile-phase additives, it can be argued that once ionic liquids are modified on the surface of materials, they no longer constitute a true ionic liquid. However, the ionic liquid offers a novel method for examining the intermolecular interactions in retention processes while simultaneously offering the potential to solve some of the most challenging problems confronting analytical separations.

**Figure 1.**
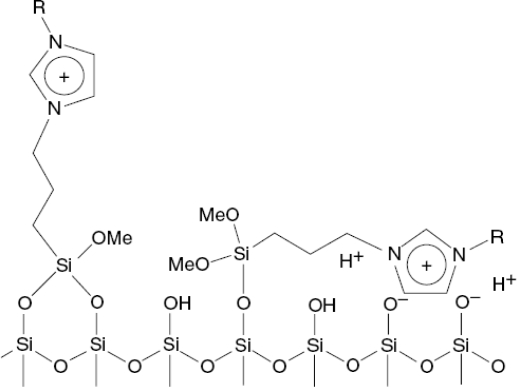
Scheme illustrating potential reorientation of bonded imidazolium ligands in response to deprotonation of residual silanols. Anion is not shown for clarity (adapted from [37]).

The earliest reports of ionic liquid-modified silica in liquid chromatography concerned the analysis of ephedrines [35] and tropane alkaloids [36]. In these studies, the *N,N*-dialkylimidazolium was attached to a 3-mercaptopropylsilane linker via a covalent bond. Retention of the analytes appeared to be governed by the hydrophobic interaction predominantly in the reversed-phase. Compared with conventional reversed-phase media, the ionic liquid-modified stationary phases provided a better peak shape and resolution while simultaneously allowing for a decrease in the organic content of the mobile phase. Colón *et al*. [37] also used a propyl linker to attach methyl- and butylimidazolium covalently to a silica support. The retention ability for organic acids was slightly higher on the butylimidazolium phase due to the differences in the hydrophobicity from the imidazolium substituent. In contrast to the increases in retention for acidic compounds with increasing pH on ion-exchange columns, the authors report a decrease in retention, which was attributed to the masking of surface silanols by reorganization of the imidazolium motif in compensation for increased silanols deprotonation (Figure 1) [37]. However, the retention of organic acids also relies on ion-exchange interactions. This phenomenon was also observed in silica-based long-chain alkylimidazolium stationary phases [38].

The retention properties of a butylimidazolium phase covalently attached to a silica support through an alkyl spacer was examined, the chromatographic retention factors for a training set of 28 small aromatic analytes were determined using a range of methanol/water [39] or acetonitrile/water [40] as mobile phases. In these studies, the linear salvation energy relationship approach was used successfully to characterize the ionic liquid-based stationary phase. The chromatographic data was fixed to multiple linear regression analysis to extract the linear solvation free energy relationship (LSFER) coefficients [41]. Plots of the experimental retention data versus the retention predicted (based on the LSFER coefficients) showed that the LSFER model with the selected compounds gave an excellent estimation of the various mobile/stationary phase interactions with the presence of ionic liquids in the stationary phase. Surprisingly, the stationary phase retained much of its reversed-phase character with the incorporation of the imidazolium cation. A comparison of the retention of a subset of the probe solutes obtained on the imidazolium column and the retention reported in the literature [41] on conventional reversed-phase columns showed that the retention characteristics of the test neutral aromatic solutes show remarkable similarity to phenyl stationary phases despite the presence of a positive charge on the new imidazolium phase. Therefore, while hydrophobic interactions are retained in the imidazolium-based phase, the presence of a cationic charge, at least some of the hydrophobic interactions with the stationary phase emanate from the imidazolium aromatic ring. Overall, the butylimidazolium column provides better discrimination between the classes of aromatic compounds in acetonitrile/aqueous mixtures than in methanol/water. Previously, in the case of the separation of imidazolium cations on phenyl phases [42], the role of *π*–*π* interactions in retention could be mediated by the addition of acetonitrile, which afforded better discrimination based on other molecular features of the selected analytes.

More recently, the emergence of a new zwitterionic stationary phase based on silica bonded to 1-alkyl-3-(propyl-3-sulfonate) imidazolium was synthesized and characterized [43]. Bases, vitamins and imidazolium ionic liquids with different alkyl chains were separated successfully on this column. The stationary phase has multiple retention mechanisms, such as anion-exchange, electrostatic attraction and repulsion interactions, as well as a hydrophobic interaction. Compared with normal imidazolium stationary phases, the appearance of a sulfonic group resulted in a new active center in the separation of samples.

## 3. Application of ILs in RP-HPLC

### 3.1. ILs used as Additives

There are many types of ILs used as additives, the most extensively studied being those based on the imidazolium cation. Table 1 lists the ILs commonly used as mobile phase additives in RP-HPLC. Some have the same cation or anion, such as [AMIM][BF_4_], [EMIM][BF_4_], [BMIM][BF_4_], [HMIM][BF_4_], and [OMIM][BF_4_]. Therefore, their properties and the additive effects on the solutes are affected by the alkyl chain. Research [44] has been carried out on the addition of the same concentrations of several ionic liquids with the same counter ion, BF_4_^−^, at different acidic pH (3.0–4.0) and concentrations of both acetonitrile (10–20%, v/v) and ionic liquid (0–10 mmol/L) to determine the effect of alkyl groups on the imidazolium ring of ionic liquids on the separation of these analytes. It is clear that the retention factors of the analytes decrease with increasing alkyl chain length, which shows that the effectiveness of these silanol-blocking agents is higher when the alkyl chain is longer. In the same study, the authors also examined the effects of the concentration of ILs. As the concentration of the ILs was increased, the effect increased initially and then decreased, which is due to a specific mechanism of the behavior of ionic liquids in the mobile phase. And that determining their effects/interactions separately is difficult and has not been reported.

In addition to the species and concentration of additives, other conditions also have important effects on the separation of solutes, such as pH. McNair *et al*. demonstrated that in a low mobile phase, where a basic analyte is fully protonated, any further decrease in pH leads to an increase in analyte retention. This effect was attributed to the influence of the acidic modifier counter anions on analyte salvation, and highest effect on the retention of basic analyte was observed at low counter anion concentrations. A further increase in the counter anion concentration caused a gradual leveling off of the retention of the basic analyte. This effect is consistent with the suggested model regarding the influence of the counter anions on analyte solvation. Because most solutes using ILs as additives were basic, it is useful to explain the retention and separation of them.

ILs were identified as a complex RP-HPLC retention mechanism involving ionic and hydrophobic interactions [1]. Under reversed-phase conditions with salt-free mobile phases, imidazolium-based ILs could be differentiated by the anion and/or cation. Mobile phases containing added salts could only differentiate ILs with different alkyl chains on the imidazolium ring. These salt-containing mobile phases could no longer separate ILs by their anions only.

Normally, the pH of the mobile phase in RP-HPLC is 7.0, which only contain water and organic solvents (methanol, acetonitrile, etc.). The pH is increased slightly when the mobile phase contains [BMIM][BF_4_] and [EMIM][BF_4_] [2]. On the other hand, the pH with [EMIM][MS] and [OMIM][MS] decreases with increasing IL concentration. This can be explained by the structure of [BF_4_]^−^ and [MS]^−^ anions in the ionic liquids. For example, the methylsulfate anion ([MS]^−^) includes a hydroxyl group.

### 3.2. Preparation of IL-Stationary Phases

Most ionic liquid-modified stationary phases for RP-HPLC were imidazolium or pyridinium functionalized silica. Generally, this type of material can be synthesized from commercial silica of spherical porous particles [45–48]. Table 3 lists some of the stationary phases that were screened.

**Figure 2.**
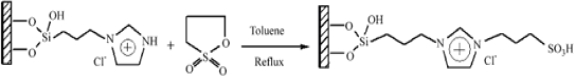
Synthesis steps used in the preparation of zwitterionic stationary phase [43].

**Figure 3.**
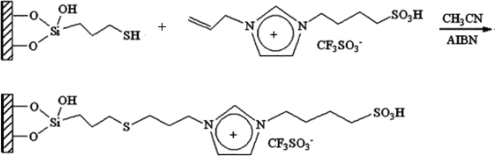
Scheme illustrating the modification of silica particles with the synthesized 1-allyl-3-(butyl-4-sulfonate)imidazolium ionic liquids [49].

The alkylimidazoles and pyridine are used widely to modify chloropropyl silica. This kind of stationary phase shows a successful application on RP-HPLC. Recently, Qiu *et al*. [43] developed a new stationary phase combining two function groups with an ionic center and a sulfonic group in a single chain. The chloropropyl silica bonded with imidazole was placed in a round-bottom flask to which anhydrous toluene and sultone were added in succession. The suspension was then refluxed with stirring. The reacted silica particles based on 1-alkyl-3-(propyl-3-sulfonate) imidazolium were then washed repeatedly with large amounts of solvents and vacuum dried (Figure 2). Another method to synthesize the bifunctional stationary phase was applied [49]. 1-Allyl-3-(butyl-4-sulfonate)-imidazolium was reacted with 3-mercaptopropyl silica in dry toluene with constant stirring. After refluxing, the reaction was stopped and washed with the solvents. The material can be obtained after drying under vacuum (Figure 3).

In addition to functionalizing ionic liquids on silica, ZrO_2_/SiO_2_^−4^ was used as a new supporter for the stationary phase [50]. The synthesis procedure was similar to the imidazolium modification of silica. The method of preparation is described in Ref. [45].

### 3.3. Separation of ILs by RP-HPLC

ILs exist in the form of ions in the column, and the effective part is cation. In order to investigate the chromatographic behavior of ILs in the column, the mixtures of ILs were separated as samples in RP-HPLC [51–53]. Ruiz-Angel *et al*. [51,52] investigated the effects of different acetonitrile/water mobile phases, different stationary phases: Kromasil C8, Zorbax Extend C18 and Zorbax Sb-Aq and various inorganic salts (NaCl, NaH_2_PO_4_, NaBF_4_, NaClO_4_ and NaPF_6_) on the separation of two mixtures of four 1-alkyl-3-methylimidazolium ionic liquids salts associated to the anions tetrafluoroborate or hexafluorophosphate, respectively. The results proved that with salt-free mobile phases, ILs differing by the anion and/or by the cation can be differentiated. When salts are added to the mobile phase, only ILs with different alkyl chain on the imidazolium ring can be separated. And the three columns gave similar separation profiles. In all cases, the retention of ILs increased with the increasing affinity of the inorganic anions for the apolar stationary phases; a phenomenon called chaotropicity. The chaotropic anion order is Cl^−^ ~ H_2_PO_4_^−^ < BF_4_^−^ ~ ClO_4_^−^ < PF_6_^−^. In the RPLC analysis of imidazolium-based IL, it is recommended to add to the mobile phase significant amounts of a salt containing a chaotropic anion. This salt addition will improve the IL peak shapes and give reproducible retention factors.

## 4. Separation of biological samples

### 4.1. Application of IL-Stationary Phases for Bioseparation

**Figure 4.**
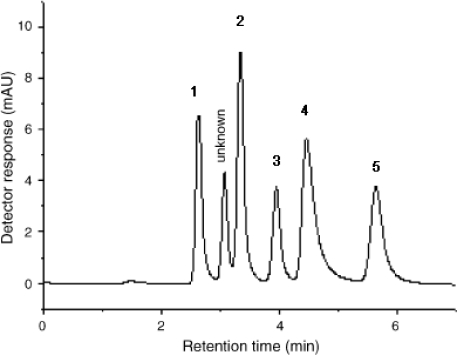
Separation of test mixtures composed of cytosine (1), thymine (2), adenine (3), 2-aminopyrimidine (4), and 6-chloroguanine (5). Mobile phase: water, detection: UV at 254 nm [55].

The ionic liquid-modified stationary phases were used gradually for bio-separation. It can be operated under normal phase conditions or reverse phase conditions. Recently, Wang *et al*. [54] separated tanshinone I and tanshinone IIA by normal phase HPLC. Although the separation of bio-compounds has been successful under normal phase conditions, it is better to apply these stationary phases to bio-separation in RP-HPLC. According to the paper [55], a mixture of cytosine, thymine, adenine, 2-aminopyrimidine and 6-chloroguanine was separated using water as the mobile phase (Figure 4).

### 4.2. Nature Plant

Ionic liquids have been used in RP-HPLC to extract the components of plants. Yao *et al*. used six types of RTILs (1-ethylpyridinium bromide ([Epy][Br]), 1-butylpyridinium tetrafluoroborate ([Bpy][BF_4_]), 1-ethyl-3-methylimidazolium bromide ([EMIM][Br]), 1-ethyl-3-methylimidazolium tetrafluoroborate ([EMIM][BF_4_]), 1-butyl-3-methylimidazolium chloride ([BMIM][Cl]) and (1-butyl-3-methylimidazolium tetrafluoroborate ([BMIM][BF_4_])) to separate octopamine, synephrine and tyramine in Citrus herbs [56]. The authors showed that the peak shapes were improved efficiently when the four 1-alkyl-3-methylimidazolium based RTILs and two *N*-alkylpyridinium-based RTILs were at the same molar concentration (32 mM) as the mobile phase additives. In addition, the alkyl chain length of the RTIL cationic component had a significant effect on the retention time of the tested basic compounds. When the butyl-based RTILs were used, the retention time of tyramine was decreased by 39% of that of the ethyl-based RTILs. For the same anion, the retention time and number of efficient plates decreased in the order of [EMIM]^+^ > [Bpy]^+^ > [BMIM]^+^. ILs have been used to separate alkaloids, 1-alkyl-3-methylimidazolium-based ionic liquids were used as mobile-phase additives to separate four ephedrines [57].

**Figure 5.**
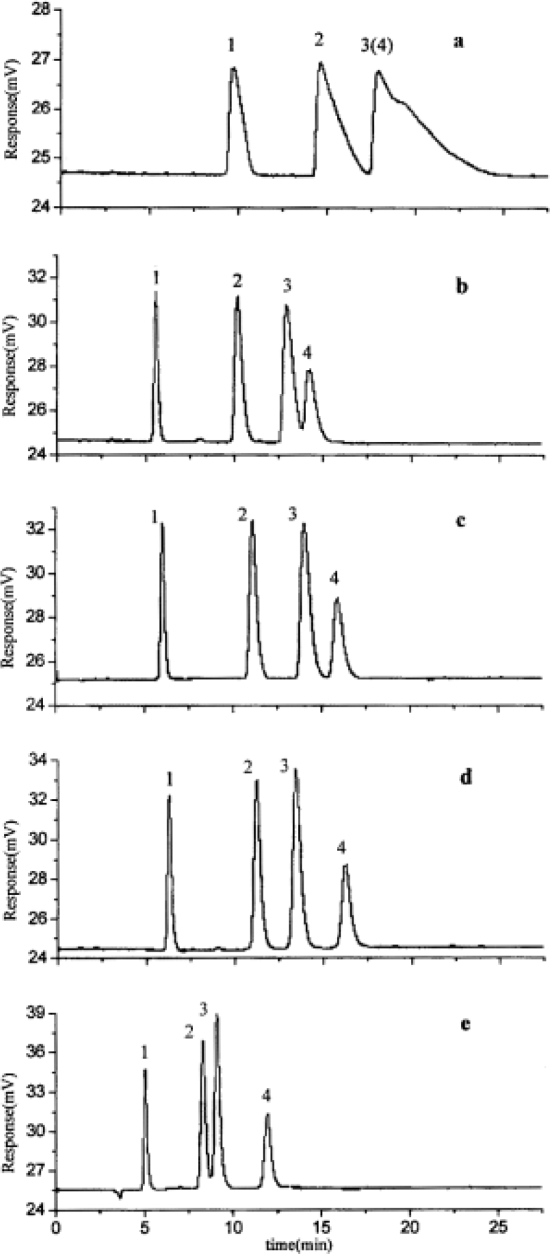
Chromatograms of ephedrines with a mobile phase containing different concentrations of [BMIM][BF4] at pH 3.0. (a) 0, (b) 2.6, (c) 5.2, (d) 20.8, and (e) 62.4 mM. Chromatographic conditions: column: C18 (5 μm, 100×4.6 mm I.D.); rate-flow: 1.0 mL/min; detection: 252 nm. Peaks: (1) NE, (2) E, (3) PE, (4) ME, [57].

Figure 5 shows the chromatograms with different concentrations of [BMIM][BF_4_] in the mobile phase. An increase in the [BMIM][BF_4_] concentration in the eluents initially causes an increase in the retention of all analytes followed by a decrease with further increases in concentration. The relationship between the concentration of [BMIM][BF_4_] and retention may be due to the following factors. When the concentrations of ionic liquids increase slightly, as shown in Figure 1, the interactions of imidazolium cations with the silanol groups on alkylsilica surface by electrostatic interaction or with the alkyl groups by hydrophobic interaction strengthen gradually, resulting in an increase in the carbon content of the stationary phase, and an increase in the retention of analytes. Because of the strong electrostatic interactions, the possibility of producing A is greater than that of producing B. With further increases in the concentration of [BMIM][BF_4_], the imidazolium cations interact with A through an electrostatic interaction, and produce a weak bilayer electronic structure that repels the basic analytes and interacts with alkyl group through a hydrophobic interaction. Therefore, the retention of analytes decreases under the repulsive and hydrophobic interactions.

### 4.3. Amines

Jiang *et al*. reported [58] the retention of catecholamines using 1-alkyl-3-methylimidazolium IL and *N*-butylpyridinium IL as mobile phase additives. The pH was adjusted to 3.0. When comparing with the one only using water, the peak symmetry of the analytes was improved greatly when four solutions of ionic liquids were used. It suggests that ionic liquids absorb on the surface of the stationary phase, and coat on the exposed silanol of the C_18_ stationary phase, which improves the peak symmetry. At the same time, the retention times of catecholamines are changed. Both 1-alkyl-3-methylimidazolium IL and *N*-butylpyridinium IL can short the retention time of catecholamines, but 1-alkyl-3-methyl-imidazolium IL is more effective and steadier than *N*-butylpyridinium IL. This shows that the retention behavior of analytes is influenced by the alkyl group of the organic cation of ionic liquids.

In another paper by Jiang *et al*. [59], some amines, including benzidine, benzylamine, *N*-ethylaniline and *N*,*N*-dimethylaniline, were analyzed by RP-HPLC. The length of the alkyl chain or counterions on different ionic liquids and their concentrations had a significant effect on the separation of these analytes. The differences between ionic liquids and tetrabutylammonium bromide on the separation of *o*-, *m*-, *p*-phthalic acids were compared. It was shown that ionic liquids are essentially ion-pair reagents, even though their hydrophobicity and hydrogen bonding also play important roles.

### 4.4. Amino Acids and Nucleic Compounds

There are many publications [60–72] reported the analysis of amino acids or nucleic compounds using ILs as additives. Mun *et al*. [60] compared the effects of [BMIM][BF_4_] on retention of three groups of l-phenylalanine and *N*-CBZ-l-phenylalanine (l-tyrosine with *N*-CBZ-l-tyrosine, l-phenylalanine with *N*-CBZ-l-phenylalanine, and l-methionine with *N*-CBZ-l-methionine). There were three primary discoveries from the results. First, the addition of an ionic liquid to the mobile phase causes a decrease in the retention behavior of l-amino acids but has little effect on *N*-CBZ-l-amino acids. A higher selectivity between l-amino acid and *N*-CBZ-l-amino acid can be achieved by increasing the ionic liquid content in the mobile phase. Second, the peak shapes of *N*-CBZ-l-methionine and *N*-CBZ-l-phenylalanine remained similar to that in the absence of an ionic liquid. However, the peak shapes of l-methionine and l-phenylalanine were transformed from asymmetric to symmetric as the ionic liquid concentration was increased. Such peak shape transformations were accompanied by peak compression, which in turn increased its maximum peak concentration and improved separation between l-amino acid and *N*-CBZ-l-amino acid. Third, the increase in feed concentration also improved the selectivity between l-phenylalanine and *N*-CBZ-l-phenylalanine. This phenomenon occurred because a high feed concentration leads to an overloaded condition for l-phenylalanine.

In the study [61], four types of ILs were used to separate four bases (cytosine, thymine, adenine, 6-chlorouracil) and four amino acids (l-histidine, l-tyrosine, l-phenylalanine, and dl-tryptophane). And the effect of the [BMIM][BF_4_] concentration was investigated primarily. An increase in the [BMIM][BF_4_] concentration in the eluents causes a decrease in retention of all analytes. The effect of the alkyl groups of the imidazolium cation showed a similar trend as other researchers [56,58] that the retention of bases decreased and the peak shapes improved with increasing alkyl chain length on the imidazolium cations. And the effect of ILs at different concentrations was investigated using [BMIM][BF_4_] and [BMIM][Cl]. [BMIM]^+^ with [BF_4_]^−^ showed superior separation of bases and amino acids to that with [Cl]^−^.

### 4.5. Drugs

Berthod *et al*. [73] compared ILs with triethylamine as mobile phase additives in the analysis of several β-blockers: acebutolol, alprenolol, labetalol, metoprolol, nadolol, pindolol and propranolol. Compared with the usual additive triethylamine, ILs showed good properties and effects. It was found to be clearly superior to the classical triethylamine additive for both efficiency and peak shape enhancement, and has little influence on the solute retention factors when decreased by triethylamine. The final advantage is that they have no influence on the mobile phase pH, which is unlike triethylamine and amines.

**Figure 6.**
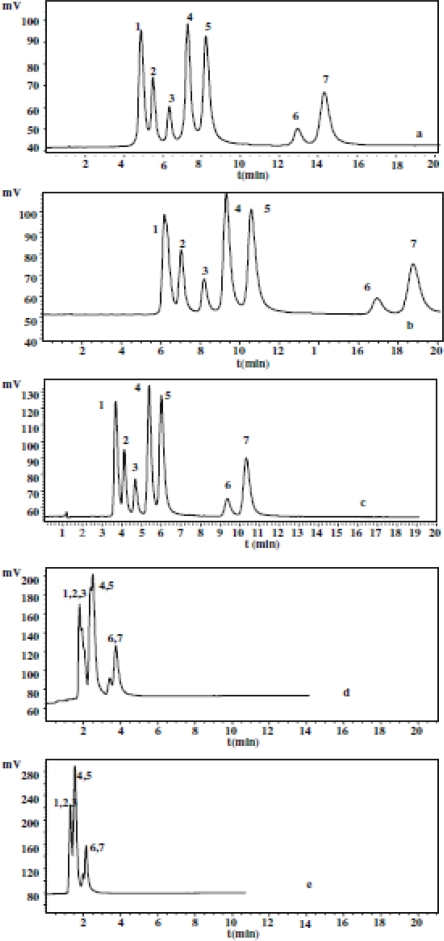
Effect of different ionic liquids on the separation of the seven antibiotics studied. Mobile phase: 10 mmol/L ammonium acetate at pH 3.0 with 13% (v/v) acetonitrile and (a) 6 mmol/L [Et4N][BF4]; (b) 6 mmol/L [EMIM][BF4]; (c) 6 mmol/L [BMIM][BF4]; (d) 6 mmol/L [HMIM][BF4]; (e) 6 mmol/L [MOIM][BF4]. Flow rate: 1 mL/min. Detection: λexc=280 nm and λem=450 nm. Peak identification: 1, FLERO; 2, CIPRO; 3, LOME; 4, DANO; 5, ENRO; 6, SARA and 7, DIFLO [74].

In reference [74], several fluoroquinolone antibiotics (i.e. fleroxacin, ciprofloxacin, lomefloxacin, danofloxacin, enrofloxacin, sarafloxacin, and difloxacin) for human and veterinary use in water samples were analyzed using five ILs with the same cation (Figure 6). The optimal separation conditions were selected, and it is the first analytical method for separating fluoroquinolone antibiotics using ionic liquids as mobile phase additives.

Three positional isomers *o, m,* and *p*-amino benzoic acid, were first separated by using the four types of ionic liquids in water-methanol. An elution order of *p > m > o* was observed with tetrafluoroborate anions, which the same sequence as that using a pure water-methanol reversed-phase system. However, the sequence was *m>p>o* in the case of methyl-sulfate anions. It was proposed that the elution order of the amino benzoic acid isomers was determined by the number of contact points available to the solute-adsorbent interactions. The pH was adjusted from 3.0 to 7.0, and the amino benzoic acid isomers were affected significantly by the pH of the mobile phase because they include amino and hydroxyl groups. When the pH of themobile phases was adjusted to 4.0, 5.0, and 6.0, every isomer existed in two forms (a molecular form and ionic form), and both forms were detected in the two peaks. When the mobile phase is acidic or approximately neutral (i.e., pH = 3.0 or pH = 7.0), the amino benzoic acid isomers will have only one molecular or ionic form. The retention factor of ionizable solutes varies considerably with pH. This shows that the addition of modifiers and the pH of the mobile phase are important.

### 4.6. Others

Alkaloids, amines, amino acids and nucleic compounds are alkaline substances, and ILs are normally used as additives to separate them. They are rarely acid substances, Jiang *et al*. first used ILs to separate acid samples: plant hormones [75]. They reported that the pH of the mobile phase played a significant role in the separation of acids, with the primary cause being hydrogen bonding on the column. Even the effect of the length of alkyl chain in ILs is different from the ones with alkaline substances; the longer the chain, the larger of the effect, but also a larger retention time.

## 5. Conclusions

Ionic liquids have applications in many fields in chemistry. They have very good selectivity towards polar and non-polar compounds, significant viscosity and many functional groups. Therefore, they have been applied as silanol-screening agents to improve the RPLC analysis of basic compounds. At low concentrations in mobile phases, the anions and cations can affect the retention time and resolution of the target compounds. The cation alkyl chain lengths affect the cation hydrophobicity. Like the use of Ils as mobile-phase additives, it can be argued that the ILs are modified on the surface of materials. Due to the hydrophobic interactions of the different cation alkyl chains, ILs can modify the surface of the silica, and new stationary phases can be used in RP-HPLC. In addition, the ion-pairing and ion-exchange of the anions and cations affect the retention times and resolution of the target compounds.

## Figures and Tables

**Table 1.** Common ionic liquids, their systematic and abbreviation names, melting points, and chemical formulas.

**Systematic name**	**Abbreviation**	**Melting Point(°C)**	**Chemical formula**	**Ref.**
1-Amyl-3-methylimidazolim tetrafluoroborate	[AMIM][BF_4_]	−88	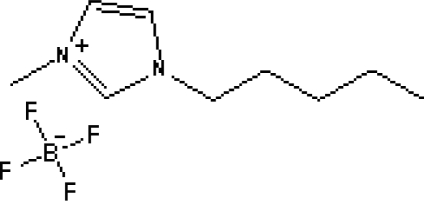	[18]
1-Butyl-3-ethylimidazolium tetrafluoroborate	[BEIM][BF_4_]	−82	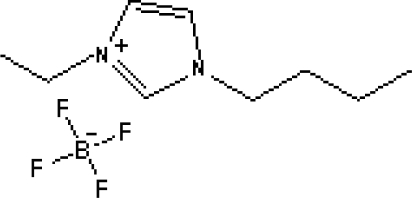	[23]
1-Butyl-3-ethylimidazolium hexafluorophosphate	[BEIM][PF_6_]	−8	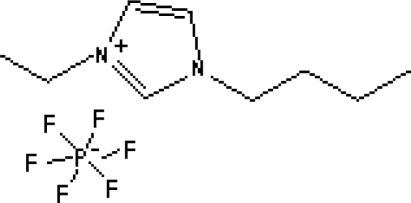	
1-Butyl-3-ethylimidazolium chloride	[BEIM][Cl]	65	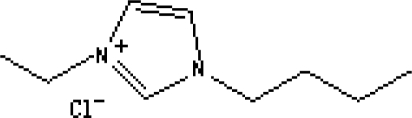	
1-Butyl-3-methylimidazolium tetrafluoroborate	[BMIM][BF_4_]	−71	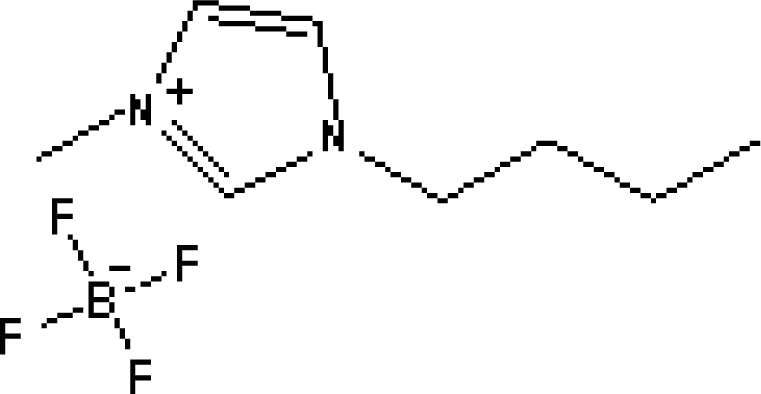	[53,54]
1-Butyl-3-methylimidazolium chloride	[BMIM][Cl]	73	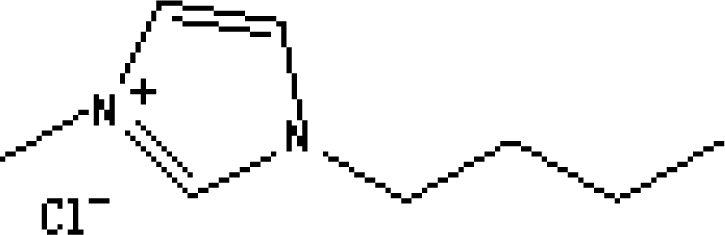	[53]
1-Butyl-3-methylimidazolium bromide	[BMIM][Br]	60	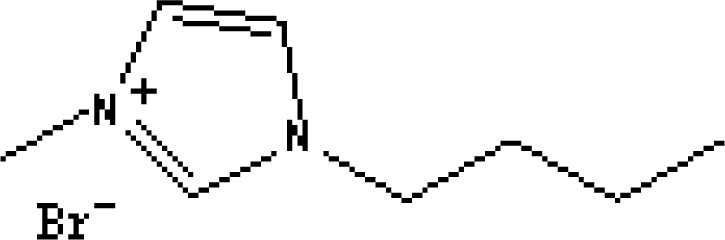	[56,73]
1-Ethyl-3-ethylimidazolium tetrafluoroborate	[EEIM][BF_4_]	15	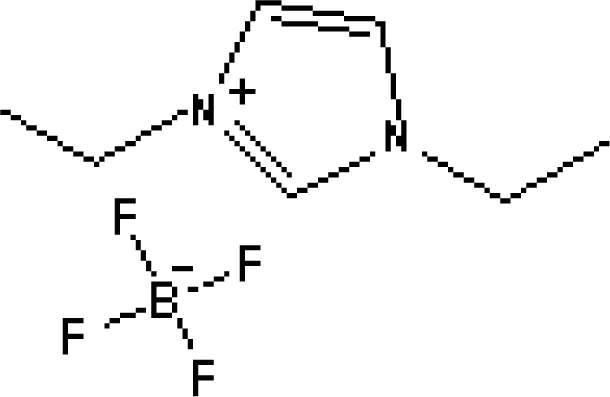	[53,54]
1-Ethyl-3-ethylimidazolium hexafluorophosphate	[EEIM][PF_6_]	58–60	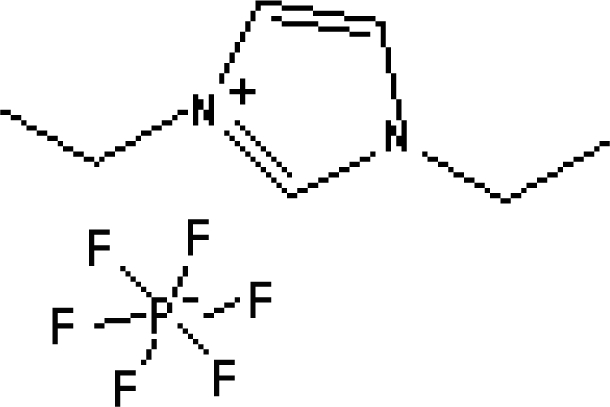	
1-Ethyl-3-methylimidazolium bromide	[EMIM][Br]	70–73	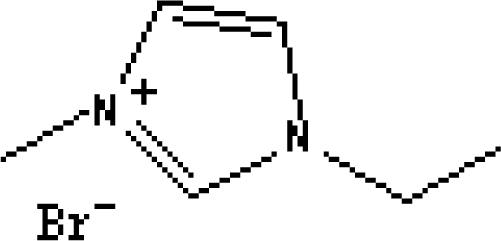	[53]
1-Ethyl-3-methylimidazolium tetrafluoroborate	[EMIM][BF_4_]	15	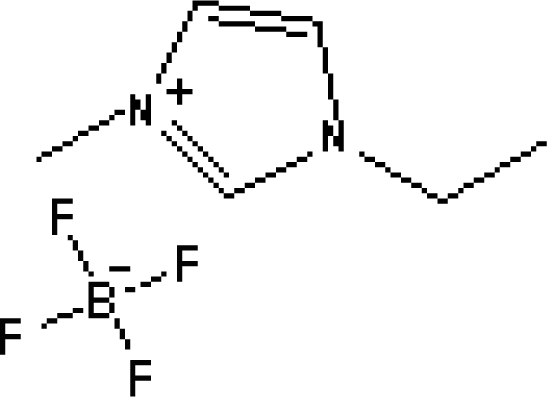	[56]
1-Hexyl-3-methylimidazolium tetrafluoroborate	[HMIM][BF_4_]	−82	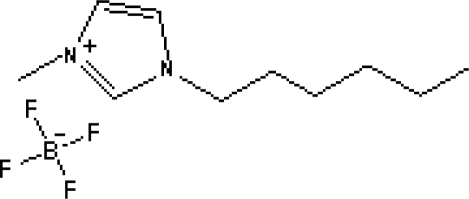	[56,73]
1-Octyl-3-methylimidazolium tetrafluoroborate	[OMIM][BF_4_]	−65	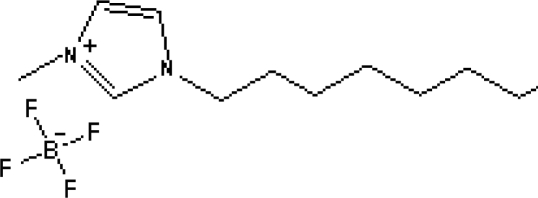	[70,73]
1-Octyl-3-methylimidazolium methyl sulfate	[OMIM][MS]	14	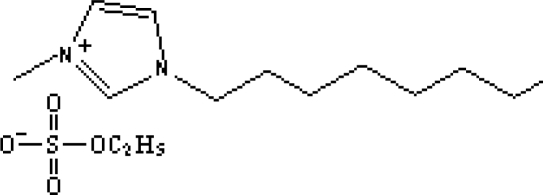	[57,63]
1-Propyl-3-methylimidazolium tetrafluoroborate	[PMIM][BF_4_]	−75	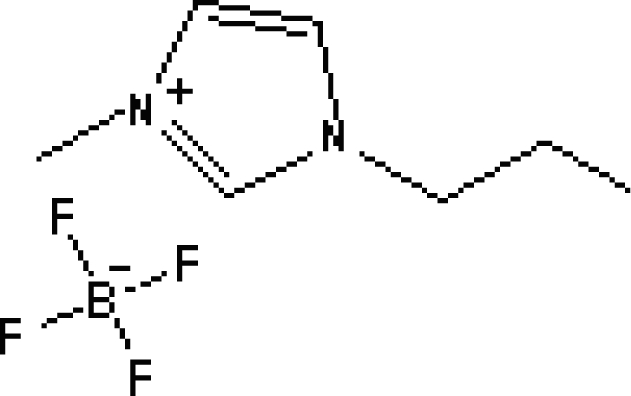	[54]

**Table 2.** Several ionic liquids building blocks.

Cation		Anion
Name	Abbreviation	

Imidazolium	[IM]	BF_4_^−^, PF_6_^−^, OH^−^
Alkypyridinium	[Pyr]	CH_3_COO^−^, COO^−^
1-Ethyl-3-hexylimidazolium	[EHIM]	NO_3_^−^, CN^−^
*N*-Ethyl-pyridinium	[NEPyr]	S_6_F_6_^−^, CF_3_SO_4_^−^,
1-Butyl-3-methylimidazolium	[BMIM]	F_6_O_4_S_6_^−^, CF_3_SO_3_^−^
1-Hexyl-3-methylimidazolium	[HMIH]	Br^−^, I^−^, Cl^−^, PhSO_3_^−^
1-Methyl-3-hexylimidazolium	[MHIH]	(CF_3_SO_2_)_2_N^−^, Al_2_C_7_^−^
1-Ethyl-3-methylimidazolium	[EMIH]	CF_3_CO_2_^−^, AlCl_4_^−^
1-Propyl-3-methylimidazolium	[PMIH]	CH_3_SO_4_^−^, CH_3_CH(OH)CO_2_^−^

**Table 3.** List of some typical stationary phases.

No.	Stationary phase	Ref.
1	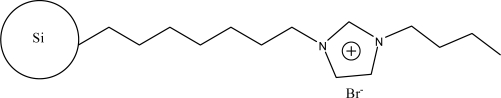	[39]
2	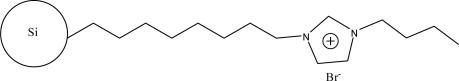	[40]
3	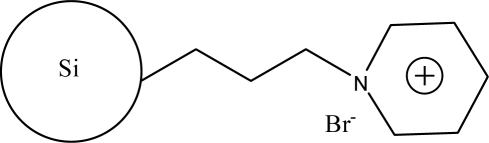	[48]
4	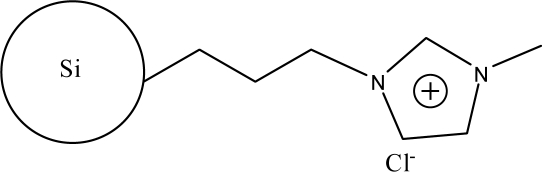	[45]
5	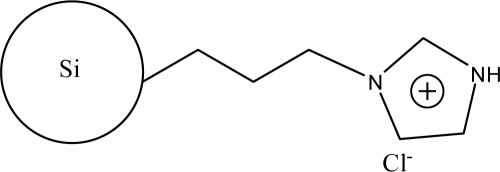	[46]
6	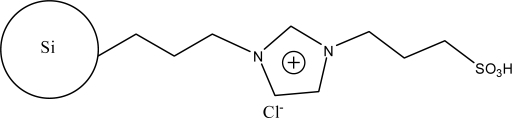	[49]
7	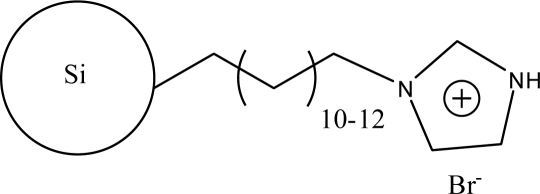	[52]
8	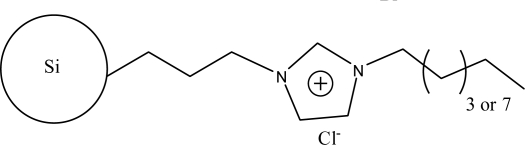	[47]
